# Supplementary material for: Evaluating the Diverse Anticancer Effects of Laos Kaempferia parviflora (Black Ginger) on Human Melanoma Cell Lines
Source: Medicina (Kaunas). 2024 Aug 22;60(8):1371. doi: 10.3390/medicina60081371 (PMC11356165; doi:10.3390/medicina60081371)
Supplement: Supplementary file 1 [file medicina-60-01371-s001.zip › medicina-3137275-supplementary.pptx]

## Slide 1
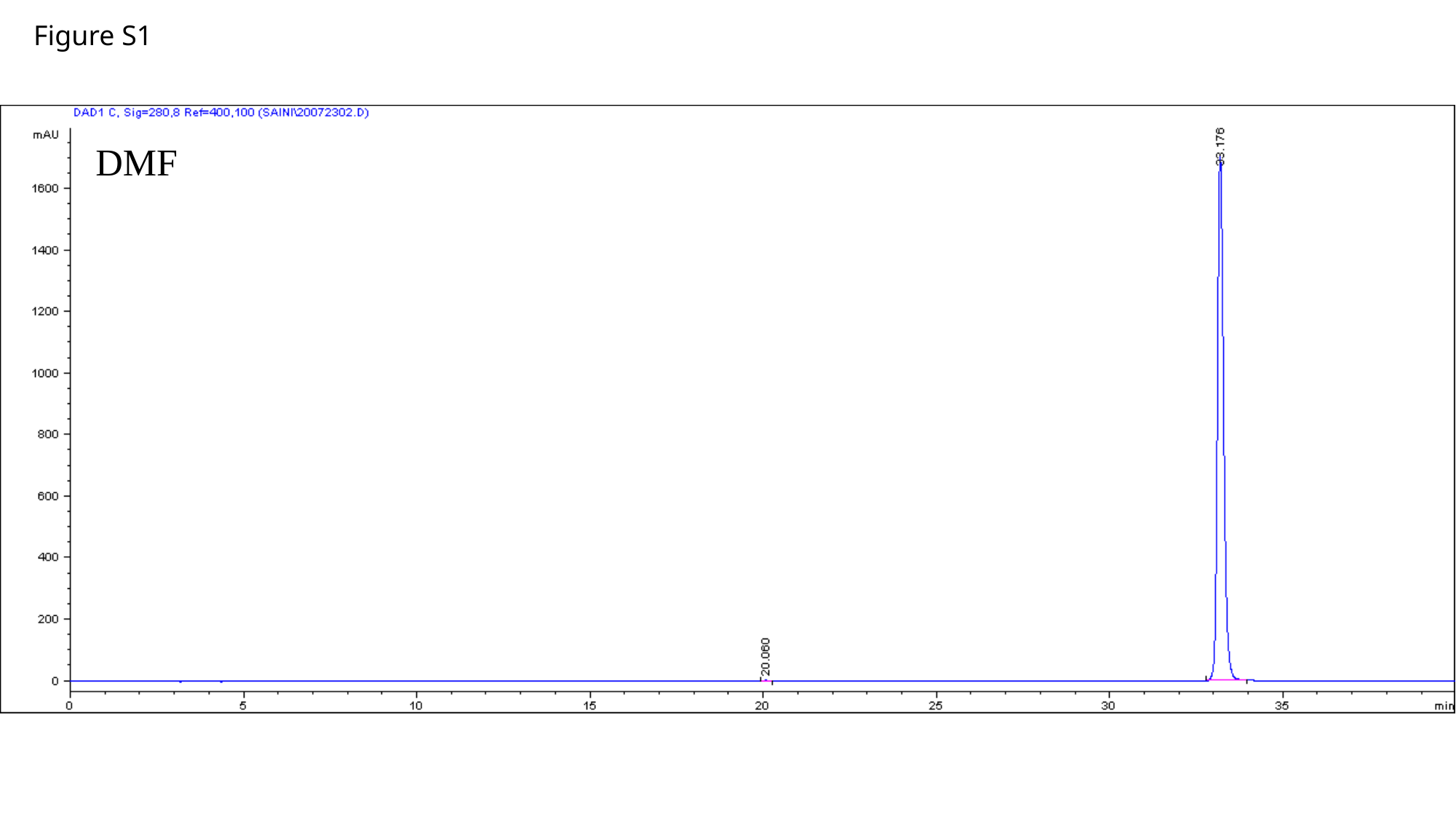

Figure S1
DMF

## Slide 2
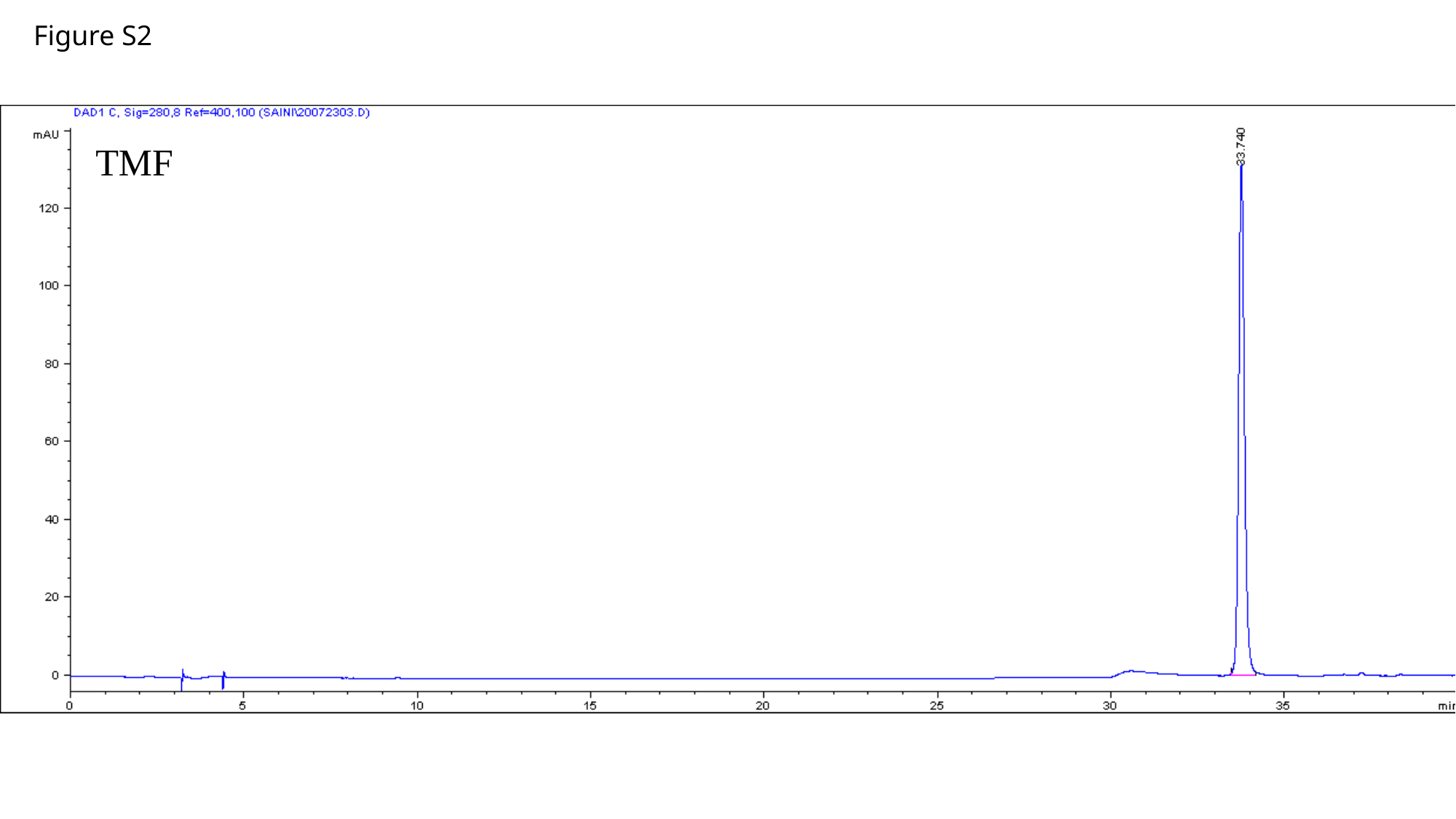

Figure S2
TMF

## Slide 3
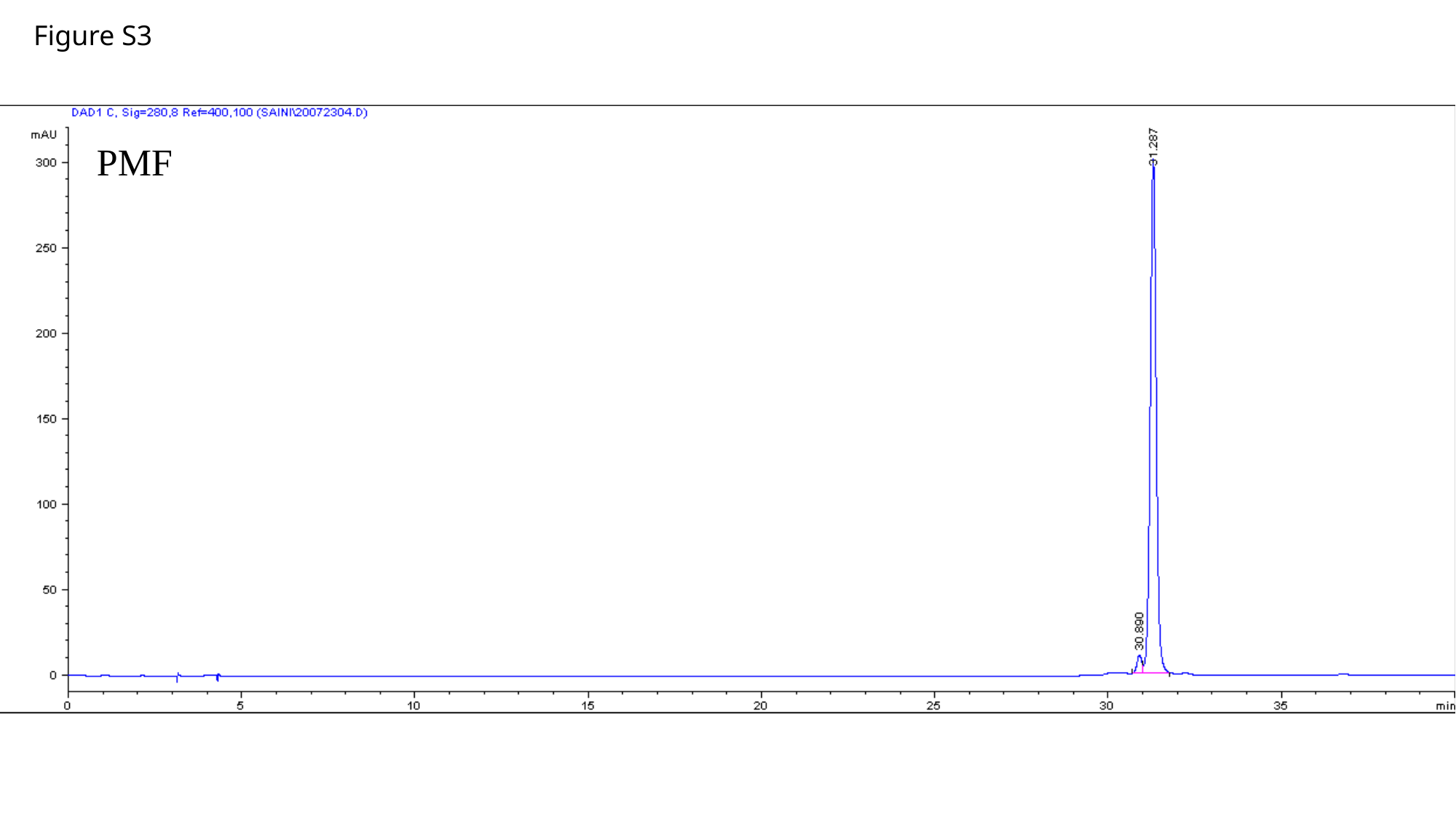

Figure S3
PMF

## Slide 4
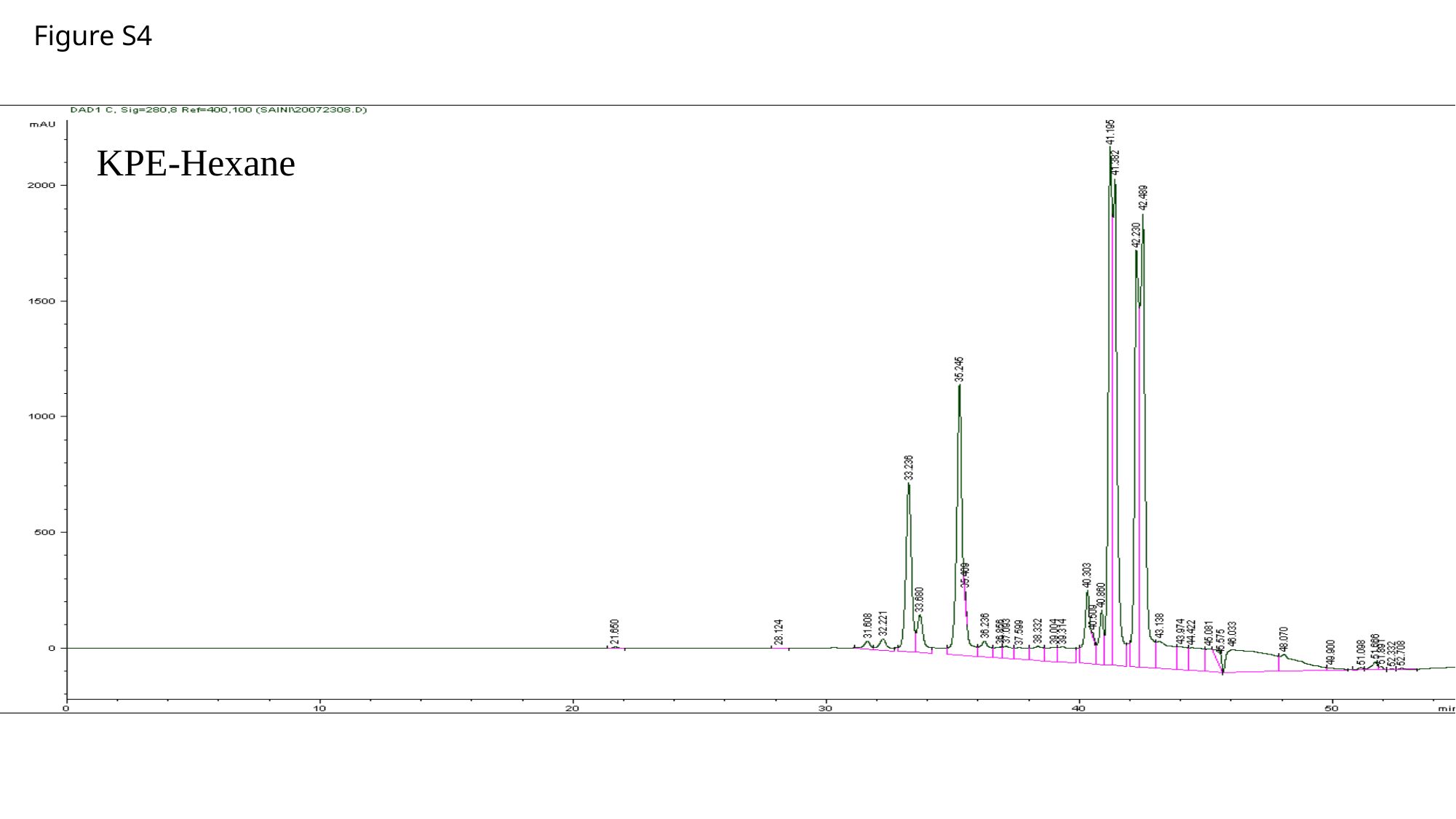

Figure S4
KPE-Hexane

## Slide 5
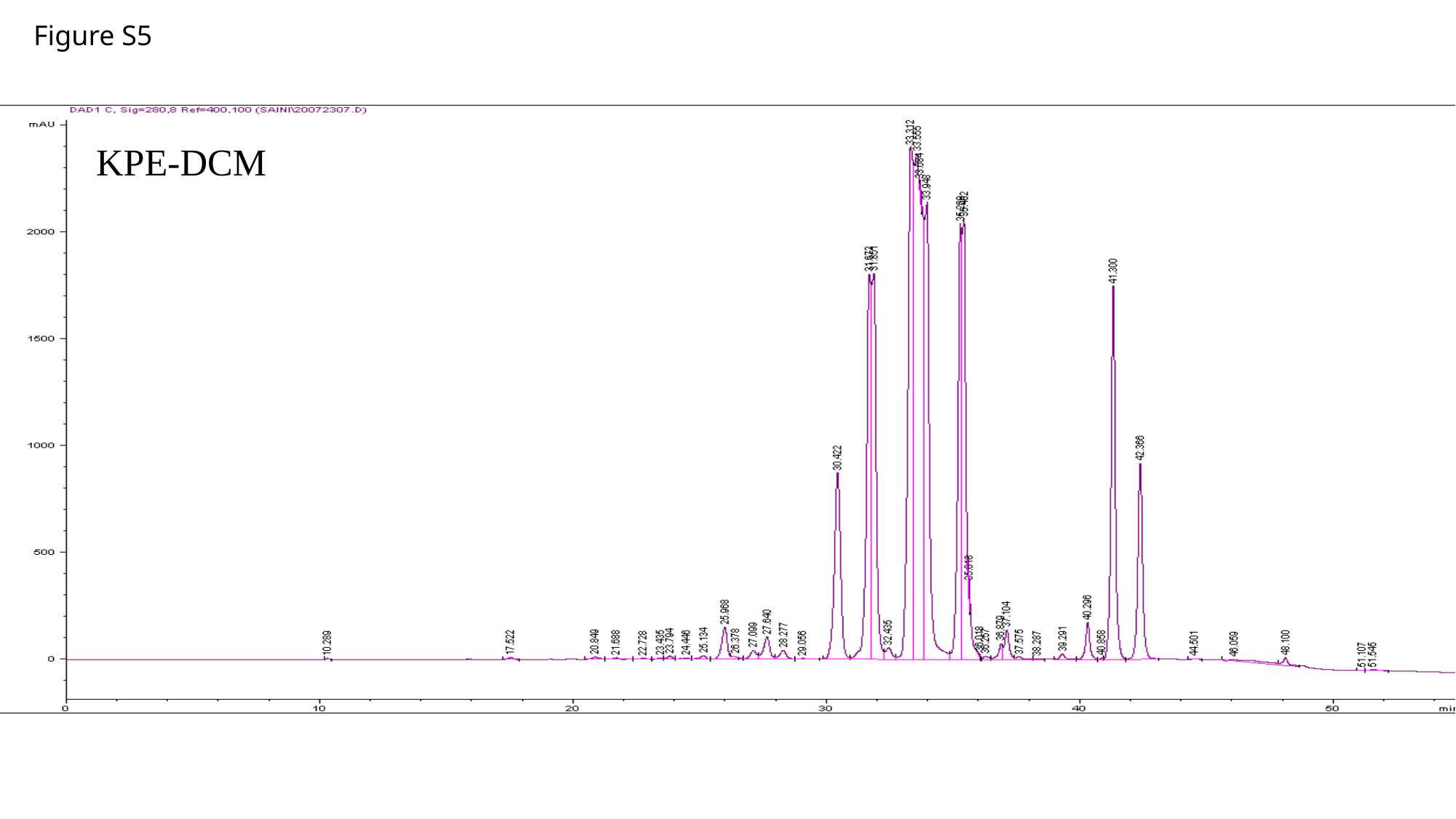

Figure S5
KPE-DCM

## Slide 6
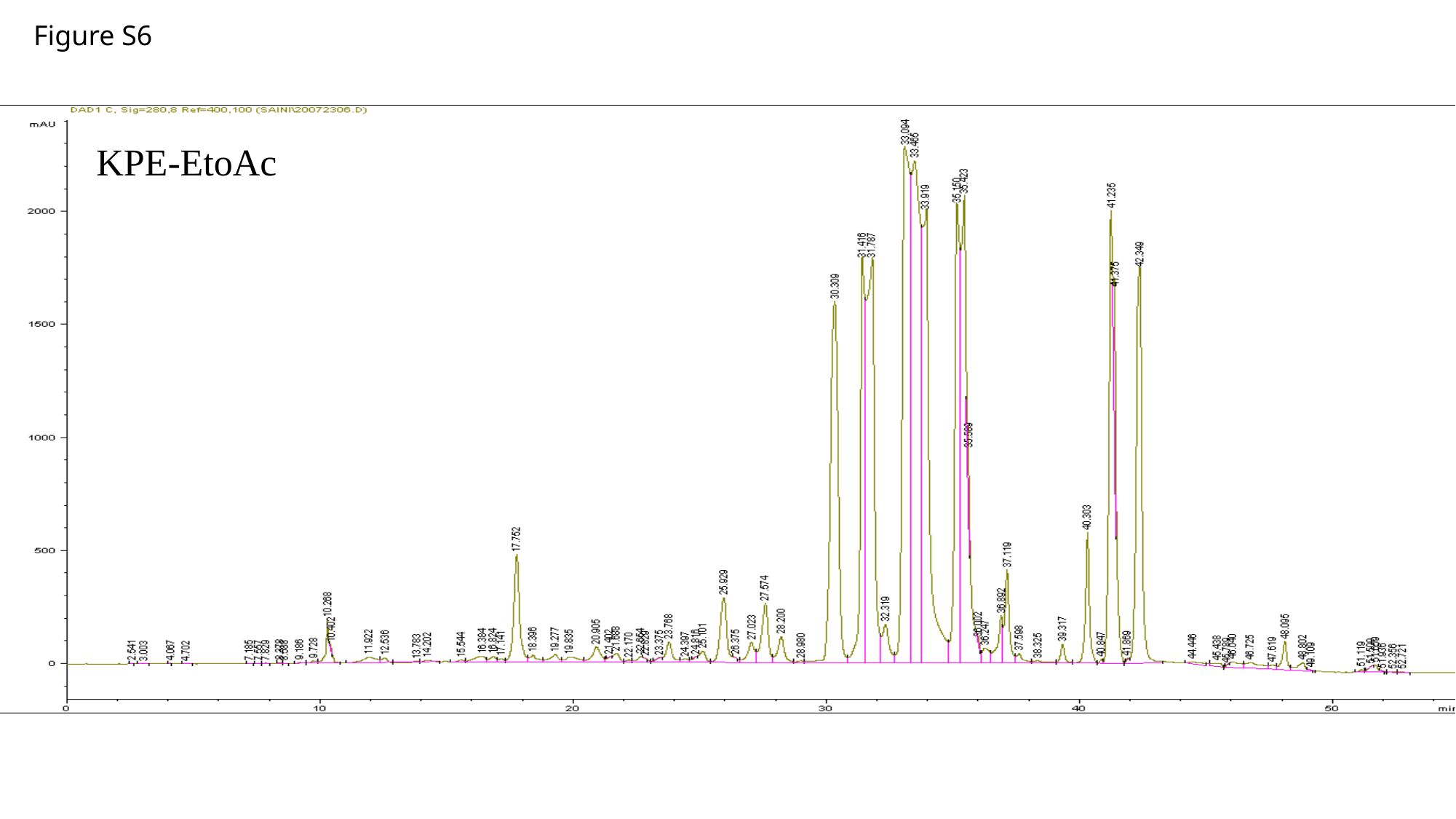

Figure S6
KPE-EtoAc

## Slide 7
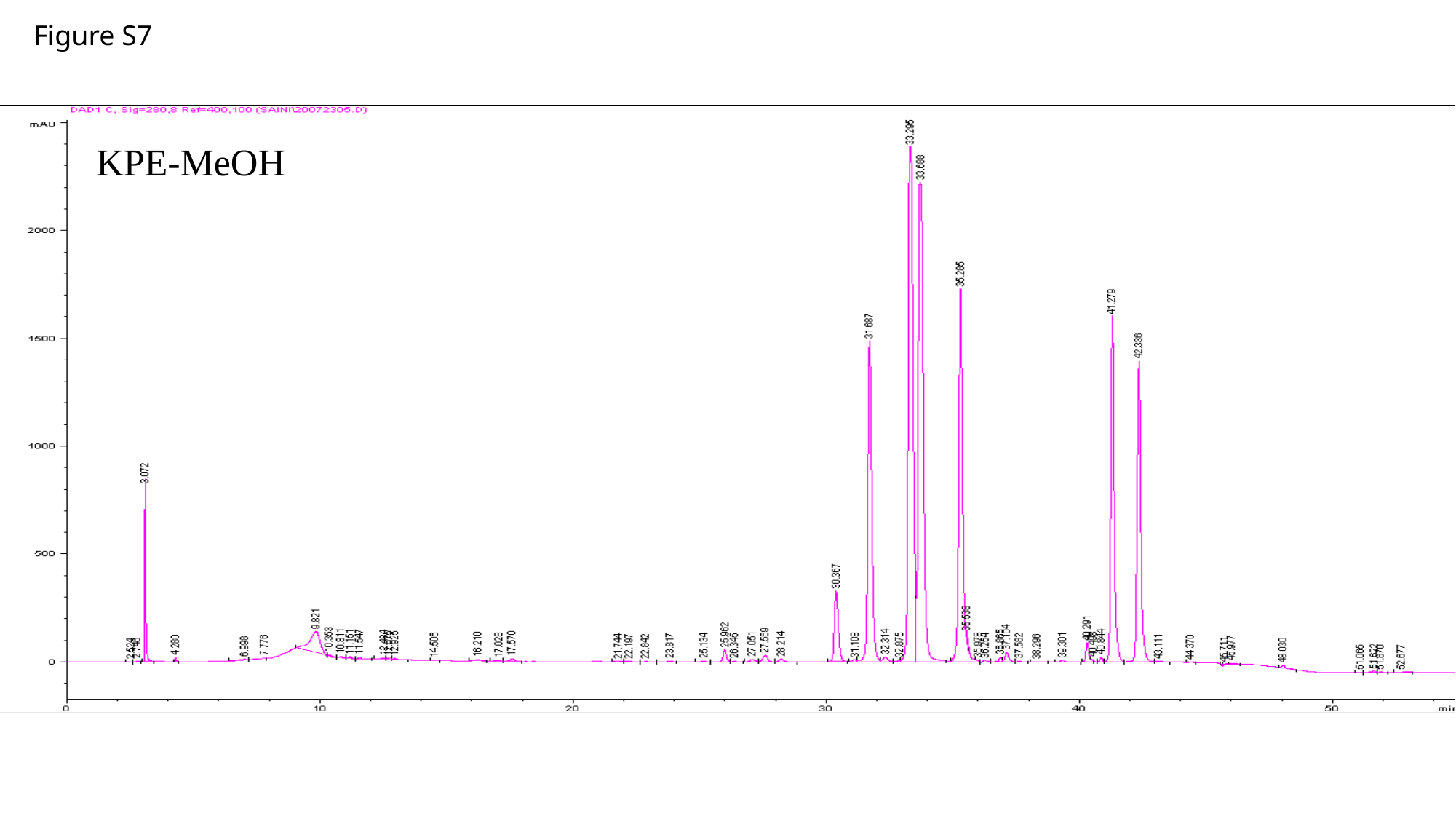

Figure S7
KPE-MeOH

## Slide 8
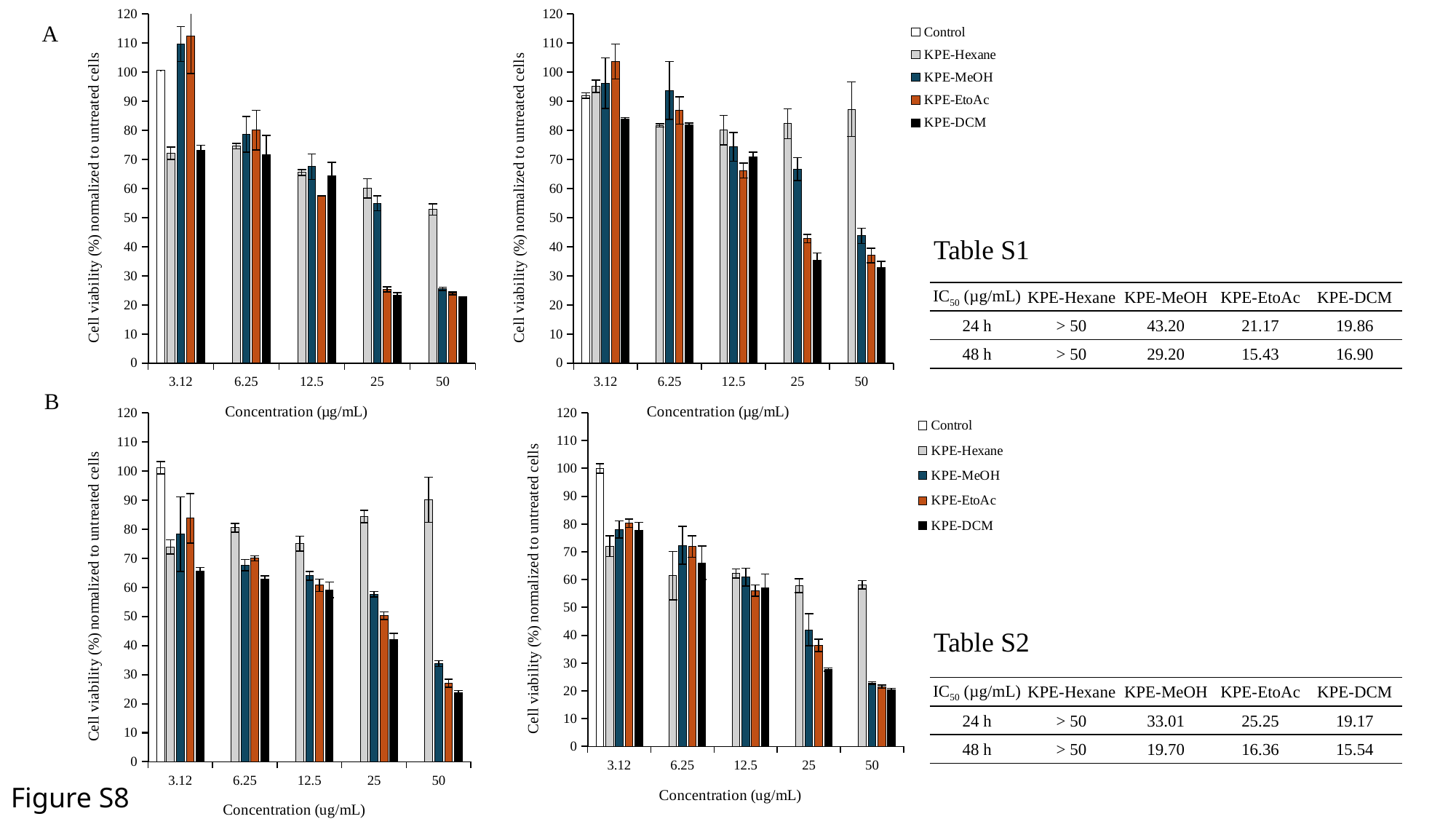

[unsupported chart]
[unsupported chart]
A
Table S1
| IC50 (µg/mL) | KPE-Hexane | KPE-MeOH | KPE-EtoAc | KPE-DCM |
| --- | --- | --- | --- | --- |
| 24 h | > 50 | 43.20 | 21.17 | 19.86 |
| 48 h | > 50 | 29.20 | 15.43 | 16.90 |
B
[unsupported chart]
[unsupported chart]
Table S2
| IC50 (µg/mL) | KPE-Hexane | KPE-MeOH | KPE-EtoAc | KPE-DCM |
| --- | --- | --- | --- | --- |
| 24 h | > 50 | 33.01 | 25.25 | 19.17 |
| 48 h | > 50 | 19.70 | 16.36 | 15.54 |
Figure S8
